# Prenatal exposure to TCDD and atopic conditions in the Seveso second generation: a prospective cohort study

**DOI:** 10.1186/s12940-018-0365-2

**Published:** 2018-02-27

**Authors:** Morgan Ye, Marcella Warner, Paolo Mocarelli, Paolo Brambilla, Brenda Eskenazi

**Affiliations:** 1 Center for Environmental Research & Children’s Health (CERCH), School of Public Health, University of California, 1995 University Avenue, Suite 265, Berkeley, CA 94720-7392 USA; 2 Department of Laboratory Medicine, University of Milano-Bicocca and Hospital of Desio, Desio-Milano, Italy

**Keywords:** Dioxin, TCDD, Tetrachlorodibenzo-p-dioxin, Allergy, Asthma, Atopy, Seveso, Prenatal exposure

## Abstract

**Background:**

2,3,7,8-Tetrachlorodibenzo-*p*-dioxin (TCDD) is a toxic environmental contaminant that can bioaccumulate in humans, cross the placenta, and cause immunological effects in children, including altering their risk of developing allergies. On July 10, 1976, a chemical explosion in Seveso, Italy, exposed nearby residents to a high amount of TCDD. In 1996, the Seveso Women’s Health Study (SWHS) was established to study the effects of TCDD on women’s health. Using data from the Seveso Second Generation Health Study, we aim to examine the effect of prenatal exposure to TCDD on the risk of atopic conditions in SWHS children born after the explosion.

**Methods:**

Individual-level TCDD was measured in maternal serum collected soon after the accident. In 2014, we initiated the Seveso Second Generation Health Study to follow-up the children of the SWHS cohort who were born after the explosion or who were exposed in utero to TCDD. We enrolled 677 children, and cases of atopic conditions, including eczema, asthma, and hay fever, were identified by self-report during personal interviews with the mothers and children. Log-binomial and Poisson regressions were used to determine the association between prenatal TCDD and atopic conditions.

**Results:**

A 10-fold increase in 1976 maternal serum TCDD (log_10_TCDD) was not significantly associated with asthma (adjusted relative risk (RR) = 0.93; 95% CI: 0.61, 1.40) or hay fever (adjusted RR = 0.99; 95% CI: 0.76, 1.27), but was significantly inversely associated with eczema (adjusted RR = 0.63; 95% CI: 0.40, 0.99). Maternal TCDD estimated at pregnancy was not significantly associated with eczema, asthma, or hay fever. There was no strong evidence of effect modification by child sex.

**Conclusions:**

Our results suggest that maternal serum TCDD near the time of explosion is associated with lower risk of eczema, which supports other evidence pointing to the dysregulated immune effects of TCDD.

**Electronic supplementary material:**

The online version of this article (10.1186/s12940-018-0365-2) contains supplementary material, which is available to authorized users.

## Background

Atopic conditions, including eczema, asthma, and hay fever, are one of the leading causes of chronic illness worldwide and are present in approximately 20% of the population [1]. The prevalence of atopic diseases has been increasing in the past few decades in both industrialized and developing countries [1]. One of the proposed reasons for this is increased exposure to environmental pollutants, as they can interfere with the immune system and subsequently alter the risk of developing allergy and asthma [2]. 2,3,7,8-tetrachlorodibenzo-*p*-dioxin (TCDD), a toxic chemical compound and a widespread environment contaminant, is produced as a byproduct of chemical and industrial processes [2, 3]. Because TCDD is highly lipophilic and has a relatively long half-life in humans of 7–9 years, it can build up in the food chain and accumulate in human fat tissues [4]. This is hazardous because TCDD is a known human carcinogen and potent endocrine disruptor, and has been shown to be immunotoxic [5–7]. For example, a broad range of immunotoxic effects have been observed in animal studies, including suppression of humoral and cell-mediated immunity, impaired infection resistance, and potential increased allergic sensitization [8–10]. Furthermore, epidemiological studies have found that exposure to dioxin or dioxin-like compounds is associated with dysregulated immune responses related to inflammation and lower risk of developing atopic conditions [11–15].

TCDD can cross the placental barrier [16] and impact development of the fetus. Because the immune system develops extensively during the fetal period, this is a critical window of susceptibility to the effects of environmental exposures. In experimental studies, prenatal and perinatal exposure to TCDD suppresses immune function and exacerbates inflammatory conditions in the offspring [17–19]. However, research on the atopic effects of in utero exposure to TCDD in humans is scarce and inconsistent [20–30]. For example, in a Danish birth cohort study, maternal serum levels of dioxin-like polychlorinated biphenyls (PCB-118) was associated with children’s risk of developing asthma [20], but not with allergic sensitization at 20 years of age [21]. In contrast, birth cohort studies in Spain and Slovakia did not find any associations in infants between dioxin-like PCBs in maternal serum and wheeze [24] and immunoglobulin E (IgE) levels [25], respectively. Overall, results from these studies are inconsistent, with some studies reporting decreased risks [22, 23, 27, 31] and others increased risk [20, 32].

Animal data also suggest that the immunomodulatory effects of prenatal exposure to TCDD may be sex-specific [19, 33]. However, few epidemiologic studies have examined sex differences in allergic conditions, and those that have showed inconsistent results. A study on Canadian pregnant women found no evidence of sex interaction between PCB-118 exposure and IgE levels in offspring [28]. In contrast, a study in a Japanese cohort of pregnant women found that dioxin concentrations in maternal blood were negatively associated with IgE levels in male infants, but not female infants [29]. When allergic conditions were examined in this cohort, there was a weak association with allergic symptoms in all infants, but the risk of infections was higher in males [30].

On July 10, 1976, a chemical plant explosion in Seveso, Italy, deposited approximately 30 kg of TCDD over the surrounding area, which was divided into exposure zones (A, B, and R) based on surface soil TCDD measurements. This accident caused one of the highest residential exposures to TCDD ever recorded [34]. A Seveso-based study found that TCDD levels in adult residents of highly contaminated and surrounding areas were negatively associated with immunoglobulin G (IgG) concentration, which suggests TCDD may play a role in suppressing antibody production [35]. In 1996, the Seveso Women’s Health Study (SWHS) was initiated to examine the effects of TCDD exposure on women’s health. SWHS is a historical cohort of women living in Seveso or nearby areas when the explosion occurred [36]. In 2014, the Seveso Second Generation Health Study was initiated to follow-up the children of the SWHS cohort who were born after the explosion or who were exposed in utero to TCDD [37].

The aim of this study is to investigate the relationship between prenatal exposure to TCDD and the risk of developing atopic conditions, including eczema, asthma, and hay fever, in the Seveso second generation cohort. We also aim to examine whether this relationship is modified by the sex of the child.

## Methods

### Study population

Details of the SWHS and the Seveso Second Generation Health Study are presented elsewhere [36, 37]. Briefly, from 1996 to 1998, participants were recruited from the female population residing in or near Seveso at the time of explosion. Women were eligible if they were 40 years old or younger, resided in the highest contaminated exposure zones (A and B) on July 10, 1976, and had archived sera collected soon after the explosion in which to measure TCDD. Of the eligible women, 981 (80%) agreed to participate. From 2014 to 2016, we conducted the Seveso Second Generation Health Study. Women from the SWHS cohort and their children born after the explosion were contacted. Eligible children included those who were born to the SWHS cohort after July 10, 1976 who were 2 years or older at follow-up. Of 950 children, 16 were deceased and 9 were less than 2 years old. Of the 925 eligible children, 677 children, 2 to 38 years of age and born to 438 mothers, were able to be reached and agreed to participate (73%). For the analysis, we excluded one child under 18 years whose mother was deceased and could not provide atopy-related information for that child, leaving a final sample of 676.

### Procedures

Participation included written informed consent for mothers and adult children (18+ years), oral assent for children ages 7–12 years, and written assent for children ages 13–17 years. The study was approved by the Institutional Review Boards of the participating institutions. All participants underwent a brief medical exam including blood pressure and anthropometry measurements, a fasting blood draw, and a personal interview. All interviews were conducted in a private room at the Hospital of Desio, Italy by trained nurse-interviewers who were blinded to the mother’s TCDD levels and zone of residence. During the interviews, adult children were asked about their reproductive and medical history, sociodemographic factors, and lifestyle characteristics. For children under the age of 18, mothers were interviewed about their children. Atopy-related questions were adapted from the International Study of Asthma and Allergies in Childhood (ISAAC) questionnaire, which has been validated and standardized for international use [38]. Cases of eczema, asthma, and hay fever were defined in two ways: 1) maternal or self-report of a diagnosis and/or symptoms related to the conditions in the past 12 months, and 2) maternal or self-report of diagnosis only.

Diagnosis of eczema was determined by a positive answer to the question, “Have you (has your child) ever had eczema?” Additional symptom-based cases were determined by positive answers to the following three questions: “Have you (has your child) ever had an itchy skin rash which was coming and going for at least 6 months?” If yes: “Have you (has your child) had this itchy rash at any time in the past 12 months?” If yes: “Has this itchy rash at any time affected any of the following places—the folds of the elbows, behind the knees, in front of the ankles, under the buttocks or around the neck, ears or eyes?”

Diagnosis of asthma was defined by a positive response to the questions: “Has a doctor ever diagnosed you (your child) with asthma?” or “Have you (has your child) ever had asthma?” Additional symptom-based cases of asthma were determined by a positive response to any of the following during the previous 12 months: a) wheezing or whistling in the chest; b) wheezing in the chest during or after exercise or physical activity; and c) dry cough at night, apart from a cough associated with a cold or chest infection.

Diagnosis of hay fever was defined by a positive response to the question, “Have you (has your child) ever had hay fever?” Additional symptom-based cases were determined by a positive response to the question, “In the past 12 months, have you (has your child) had a problem with sneezing or a runny or blocked nose when you (your child) did not have a cold or the flu?”

### Laboratory analysis

Details of the serum sample collection and TCDD concentration measurements are presented elsewhere [36, 39]. Maternal serum was collected soon after the explosion, and TCDD was measured in archived sera of adequate volume by high-resolution gas chromatography/high-resolution mass spectrometry methods at the U.S. Centers for Disease Control and Prevention [40]. Non-detectable TCDD values were assigned one-half of the detection limit. For this study, the median limit of detection was 18.8 ppt, lipid-adjusted [41]. We estimated maternal TCDD levels at pregnancy by extrapolation from the nearest TCDD measurement in 1976, 1996, or 2008 using a first-order kinetic model with a half-life that varies with initial dose, age, and covariates as described previously [37, 42]. TCDD values are reported on a lipid–weight basis as picograms per gram lipid or parts per trillion (ppt) [43].

### Statistical analysis

Prenatal TCDD exposure was examined in two ways: 1) maternal initial (1976) serum TCDD level and 2) estimated TCDD at pregnancy. Initial TCDD levels were assessed to examine whether the primary dose permanently impacts the woman’s endocrine or reproductive system, resulting in heritable changes to her children. TCDD levels at pregnancy were assessed to determine whether direct fetal exposure during pregnancy impacts the child’s atopic outcomes. Because of the skewed distribution of TCDD values, the maternal 1976 TCDD and estimated TCDD at pregnancy variables were log10-transformed for all analyses. Lipid-adjusted values of TCDD concentration were analyzed as continuous variables. We fitted generalized additive models (GAMs) to explore the shape of the relationships between the exposures and outcomes. After transformation, the functions were sufficiently linear and no further transformation was necessary. Eczema, asthma, and hay fever were analyzed as binary variables (present vs. not present).

Potential covariates and confounders were identified a priori based on a directed acyclic graph (DAG) and existing literature on immune function, asthma, and allergic diseases [44–48]. Maternal variables considered included age at explosion and atopic history. Child-based variables included maternal age at pregnancy, maternal body mass index (BMI) closest to birth year, maternal smoking during pregnancy, pre-term birth, season of birth, breastfeeding duration, birth order, child sex, child age, and primary wage earner education status. Potential confounding variables from the DAG were considered for the regression models if they were significantly associated with at least one of the outcome variables in bivariate analyses (*P* < 0.2). Final models were adjusted for maternal age at explosion, atopic history, age at pregnancy, BMI closest to birth year, and smoking during pregnancy; child birth order, sex, and age; and primary wage earner education at follow-up.

Multivariable log-binomial regressions were used to examine the relationship between maternal TCDD levels and the risk of eczema, asthma, and hay fever. For models that were not able to converge using log-binomial regressions, Poisson regressions with a robust variance estimator were used [49]. These included the models that examined the association between TCDD estimated at pregnancy and hay fever defined by both symptomology and diagnosis. For all of the models, a clustered sandwich estimator of variance was used to account for non-independence of sibling clusters (*n* = 438 clusters) [50]. We also tested for effect modification by child sex in all models by including a cross-product term between the exposures and child sex. In sensitivity analyses, we repeated final models for asthma, excluding one person who self-reported a diagnosis of asthma, but did not report that it was doctor diagnosed. We also repeated final models for eczema, excluding one child who reported “don’t know” rather than “no” for self-report of a diagnosis of eczema. All statistical analyses were conducted using Stata, version 13 [51]. Statistical significance was set to be *P*-values < 0.05, unless otherwise specified. Interaction *P*-values < 0.2 were considered to be significant.

## Results

Demographic and selected characteristics of our study sample are presented in Table 1. For the 438 mothers, the average age at explosion was 14.7 (standard deviation (SD) = ±8.0) years, and the average age at pregnancy was 29.8 (SD = ±5.1) years; 20.8% reported a history of asthma or allergy. Of the 676 children, 7.5% had mothers who were obese near the time of birth, 9.3% had mothers who smoked during pregnancy, 6.8% were born pre-term, 31.4% were breastfed for five or more months, 59.3% were first-born, and 50.3% were female. At interview, the average age of the children was 22.9 (SD = ±9.9) years, and 70.4% of the household primary wage earners had achieved an education of high school or greater. The distribution of births for each birth season was approximately equal (Table 1).

**Table 1:** Distribution of maternal 1976 serum TCDD levels (ppt, lipid-adjusted) by maternal and child-based characteristics; SWHS, Italy, 1976–2016

	N (%) *N* = 676 children of 438 mothers	1976 Serum TCDD (ppt) Median (IQR)
Maternal characteristics (*n* = 438)		
Age at explosion (years)*		
< 11	137 (31.3)	162.0 (57.8–327.0)
11–17	143 (32.7)	55.0 (26.7–111.0)
18+	158 (36.1)	43.8 (22.0–99.5)
Atopic history		
Yes	91 (20.8)	85.2 (35.0–222.0)
No	347 (79.2)	63.3 (29.1–166.0)
Age at pregnancy (years)		
< 26	140 (20.7)	44.1 (21.0–103.5)
26–29	194 (28.7)	62.3 (29.6–160.0)
30–33	177 (26.2)	76.2 (34.4–214.0)
34+	165 (24.4)	76.9 (37.5–214.0)
BMI (kg/m^2^) closest to birth year		
< 25 (underweight/normal)	477 (70.6)	67.5 (30.8–185.0)
25–29 (overweight)	148 (21.9)	49.9 (23.7–164.0)
30+ (obese)	51 (7.5)	86.7 (28.4–162.0)
Smoking during pregnancy ^a^		
Yes	63 (9.3)	55.2 (20.7–109.0)
No	609 (90.1)	66.8 (30.2–185.0)
Child Characteristics (*n* = 676)		
Pre-term birth (< 37 weeks)		
Yes	46 (6.8)	90.2 (33.2–245.0)
No	630 (93.2)	64.3 (29.6–173.0)
Season of birth		
Spring	174 (25.7)	55.5 (29.0–196.0)
Summer	167 (24.7)	66.8 (29.9–174.0)
Fall	171 (25.3)	66.7 (28.6–176.0)
Winter	164 (24.3)	72.6 (33.4–170.0)
Breastfeeding duration (months)		
< 1	214 (31.7)	60.3 (28.6–150.0)
1–4	250 (37.0)	69.9 (30.6–173.0)
5+	212 (31.4)	66.2 (34.4–212.0)
Birth order		
1	401 (59.3)	70.0 (31.0–185.0)
2	236 (34.9)	63.3 (29.3–172.0)
3+	39 (5.8)	49.9 (19.5–105.0)
Sex		
Males	336 (49.7)	60.1 (26.9–158.5)
Females	340 (50.3)	75.0 (33.2–196.0)
Age at follow-up*		
2–13	161 (23.8)	166.0 (49.0–433.0)
14–22	167 (24.7)	66.2 (33.2–163.4)
23–30	171 (25.3)	60.0 (29.4–123.0)
31–39	177 (26.2)	40.7 (21.3–85.2)
Primary wage earner education at follow-up		
Less than high school	200 (29.6)	65.3 (29.8–153.5)
High school	251 (37.1)	75.2 (33.3–247.0)
Greater than high school	225 (33.3)	60.0 (23.9–140.0)

^a^Numbers do not add to 100% because of missing data. 4 missing values for maternal smoking during pregnancy

*p < 0.05 (ANOVA significant difference in log_10_TCDD by covariates)

The median lipid-adjusted serum TCDD concentration near the time of the explosion for the 438 mothers was 64.7 ppt (interquartile range (IQR): 29.9–179.0), and the median estimated TCDD concentration at pregnancy was 11.2 ppt (IQR: 5.3–29.9). Consistent with our previous report, mothers who were youngest at the time of explosion had the highest median initial TCDD concentration (Table 1) [39].

Among the 676 children with known atopy status, there were 98 eczema, 152 asthma and 243 hay fever cases from maternal and self-report of diagnosis or symptoms. The average age at diagnosis was 10 years for eczema (*n* = 53), 7 years for asthma (*n* = 77), and 11 years for hay fever (*n* = 126). Compared to non-cases, children with eczema were more likely to have mothers who had a history of asthma or allergy. Females were less likely than males to have asthma. Children who were first born, had mothers with a history of asthma or allergy, and lived with a primary wage earner who was less educated were more likely to have hay fever (data not shown).

Table 2 presents the associations between maternal 1976 TCDD levels and eczema, asthma, and hay fever. Maternal 1976 TCDD was not associated with eczema (adjusted RR per 10-fold increase in TCDD = 0.93, 95% CI: 0.69, 1.27). When we limited the analysis to cases defined only by reported doctor diagnosis (*n* = 55), the results showed a significant inverse association between 1976 TCDD and eczema (adjusted RR = 0.63, 95% CI: 0.40, 0.99) (Table 2). Maternal 1976 TCDD was not associated with asthma or hay fever by either case definition. Unadjusted results are presented in Additional file 1: Table S1.

**Table 2:** Adjusted^a^ regression models of associations of maternal 1976 TCDD levels with atopic conditions in children, SWHS, Italy, 1976–2016

		Adjusted RR (95% CI)			
Outcomes	Cases N (%)	Total	Male	Female	p-interaction
Eczema					
Diagnosis and symptoms	98 (14.5)	0.93 (0.69, 1.27)	1.01 (0.72, 1.41)	0.86 (0.53, 1.37)	0.54
Diagnosis only	55 (8.2)	0.63 (0.40, 0.99)	0.64 (0.41, 0.99)	0.62 (0.29, 1.32)	0.96
Asthma					
Diagnosis and symptoms	152 (22.5)	0.93 (0.71, 1.21)	0.90 (0.65, 1.24)	0.97 (0.66, 1.43)	0.72
Diagnosis only	81 (12.0)	0.93 (0.61, 1.40)	1.04 (0.65, 1.66)	0.77 (0.42, 1.40)	0.38
Hay fever					
Diagnosis and symptoms	243 (36.0)	1.02 (0.86, 1.20)	0.91 (0.73, 1.13)	1.20 (0.95, 1.52)	0.07
Diagnosis only	130 (19.3)	0.99 (0.76, 1.27)	0.94 (0.67, 1.32)	1.04 (0.73, 1.49)	0.67

^a^Models adjusted for maternal age at explosion, atopic history, age at pregnancy, BMI closest to birth year, smoking during pregnancy, birth order, child sex, child age, and primary wage earner education

Table 3 presents the associations between TCDD estimated at pregnancy and eczema, asthma, and hay fever. We found no evidence of a significant association between TCDD estimated at pregnancy and eczema (adjusted RR per 10-fold increase in TCDD = 0.90, 95% CI: 0.60, 1.38), asthma (adjusted RR = 0.93, 95% CI: 0.65, 1.34), or hay fever (adjusted RR = 1.09, 95% CI: 0.90, 1.31). All associations remained non-significant when we limited the samples to include cases defined by a diagnosis only (Table 3).

**Table 3:** Adjusted^a^ regression models for associations of TCDD estimated at pregnancy with child atopic conditions, SWHS, Italy, 1976–2016

		Adjusted RR (95% CI)			
Outcomes	Cases N (%)	Total	Male	Female	p-interaction
Eczema					
Diagnosis and symptoms	98 (14.5)	0.90 (0.60, 1.38)	0.86 (0.48, 1.53)	0.95 (0.60, 1.51)	0.75
Diagnosis only	55 (8.2)	0.71 (0.45, 1.12)	0.79 (0.46, 1.38)	0.61 (0.32, 1.19)	0.52
Asthma					
Diagnosis and symptoms	152 (22.5)	0.93 (0.65, 1.34)	0.84 (0.54, 1.33)	1.07 (0.72, 1.60)	0.34
Diagnosis only	81 (12.0)	0.92 (0.52, 1.63)	0.77 (0.40, 1.45)	1.22 (0.62, 2.40)	0.21
Hay fever					
Diagnosis and symptoms	243 (36.0)	1.09 (0.90, 1.31)	1.06 (0.84, 1.34)	1.13 (0.88, 1.45)	0.67
Diagnosis only	130 (19.3)	1.01 (0.75, 1.36)	0.96 (0.65, 1.41)	1.07 (0.72, 1.58)	0.67

^a^Models adjusted for maternal age at explosion, atopic history, age at pregnancy, BMI closest to birth year, smoking during pregnancy, birth order, child sex, child age, and primary wage earner education

There was limited evidence of effect modification by child sex on the relationship between maternal 1976 TCDD or TCDD estimated at pregnancy and atopic conditions. Specifically, among the samples that included cases defined by both a diagnosis and symptomatology, we found no evidence of effect modification by child sex on maternal 1976 TCDD and eczema (p-interaction = 0.54) or asthma (p-interaction = 0.72). However, there was evidence of effect modification by child sex for hay fever (*p* = 0.07) with non-significantly increased risk among female children (adjusted RR = 1.20, 95% CI: 0.95, 1.52) but not male children (adjusted RR = 0.91, 95% CI: 0.73, 1.13) (Table 2). However, there was no evidence of effect modification by sex on TCDD estimated at pregnancy and eczema (p-interaction = 0.75), asthma (p-interaction = 0.34), or hay fever (p-interaction = 0.67) (Table 3). Similarly, among the samples that included only diagnosis-based case definitions, we found no evidence of effect modification by child sex on maternal 1976 TCDD or TCDD estimated at pregnancy and atopic conditions (Tables 2 and 3).

In sensitivity analyses, we repeated the final models for asthma, excluding the one asthma case that was not doctor diagnosed, and also repeated the final models for eczema, excluding one child who reported “don’t know” for their condition status. The results were similar (data not shown).

## Discussion

In this analysis of children of mothers who were exposed to TCDD in Seveso, Italy in 1976, we found no evidence of an association between maternal 1976 serum TCDD or TCDD estimated at pregnancy and asthma or hay fever although there was a non-significantly higher risk in females than males in some analyses. However, we observed a significant inverse association between eczema and initial 1976 TCDD, and a non-significant inverse relationship with eczema and TCDD estimated at pregnancy, with no strong evidence of differences by sex.

The decreased risk of eczema is consistent with three previous studies. A study in the Netherlands found that both prenatal and postnatal exposure to dioxin concentrations measured in breast milk had a non-significant inverse relationship with eczema [31]. Two studies in Norway also reported a non-significant inverse relationship between maternal dietary exposure to dioxins and dioxin-like polychlorinated biphenyls (PCBs) at the 22nd week of gestation and the risk of eczema and itchiness in the face and joints [22, 23].

However, in contrast to our null findings of TCDD and asthma symptomatology and allergy, one of the Norwegian studies found an increased risk of wheeze [23], and a Japanese cohort study of 18-month olds found an increased risk for maternal report of food allergies associated with polychlorinated dibenzo-*p*-dioxins (PCDD), polychlorinated dibenzofurans (PCDF), and dioxin-like PCBs measured in maternal blood in the third trimester [30]. While these two studies did find an increased risk, the children in the Norwegian study were one-year-old, and most wheeze symptoms subside when children reach ages three to five [23], and the Japanese study measured food allergies, which may be related to food intolerance instead of the immune system or atopic conditions [30]. On the other hand, a Dutch study of four-year-olds found a decreased risk of asthma-related symptoms associated with prenatal exposure to dioxin-like PCBs [27], which persisted to school-age [26], but there was no association found for total dioxin toxic equivalents (TEQ). Another Dutch study of older children with prenatal and postnatal exposure to dioxins measured in the mother’s breast milk shortly after birth also reported a decreased risk in allergies [31]. The levels of dioxin are lower in these studies [27, 31] than in the Seveso cohort [40, 43], and some studies measured a mixture of PCBs, which also included non-dioxin-like compounds [26], while others measured a combination of PCDDs, PCDFs, and other dioxin-like compounds [26, 27, 30, 31].

The decreased risk of eczema can be explained by immunomodulatory effects of TCDD [52]. Although a decrease in eczema appears beneficial, the mechanisms underlying this occurrence may be related to other immunotoxic effects of TCDD, such as diminishing the mobilization of immune cells or suppressing adaptive immune responses, including some eczemas. The influence of TCDD on immunoregulation has been widely proposed to be related to its interaction with the aryl hydrocarbon receptor (AhR). TCDD is a potent AhR ligand, and its activation of the receptor alters many downstream signals that shape immune response. For example, TCDD exposure may interfere with the balance of regulatory T (Treg) cells and Type 1 (TH1) and Type 2 (TH2) helper T cells [52]. These cells influence other immune cells’ response to antigens, such as initiating the production of immunoglobulin E (IgE) antibodies that cause allergic reactions. AhR-mediated responses with TCDD may expand Treg and TH1 helper cell populations, while suppressing TH2 cell immune responses [52, 53]. Another AhR-mediated mechanism by which TCDD may impact eczema risk is by affecting the skin barrier. In mice, in utero exposure to TCDD increased epidermal barrier formation via activation of the AhR [54] and increased expression of the filaggrin gene; a deficiency and mutation in filaggrin has been associated with eczema [55]. In vitro studies also show that TCDD exposure accelerates keratinocyte differentiation [56]. Therefore, there may be an interplay of dermatological and immunomodulatory responses from TCDD’s interaction with AhR, resulting in lower risk of eczema.

The immunomodulatory effects of prenatal and perinatal TCDD exposure have also been reported in animal studies. For example, hematopoietic progenitor cells from offspring of rodents exposed to TCDD had decreased B and T-lymphocyte differentiation capacity, compared to offspring of unexposed rats [17, 57]. In mice, male offspring of dams exposed to TCDD during both gestation and lactation exhibited a decrease in spleen weights and interleukin-4 production of splenic immune cells [33]. Another study of perinatally-exposed rats reported similar findings of decreased thymus/body weight ratio, delayed-type hypersensitivity response, and percentage of splenic CD3+/CD4 − CD8− cells, which are signs of attenuated cell-mediated immunity [18]. Furthermore, mice perinatally exposed to TCDD and sensitized with injections of ovalbumin exhibited lower serum levels of allergen-specific IgE, indicating that perinatal exposure to TCDD may lead to decreased allergic sensitization [58].

An explanation for why we did not observe significant associations in asthma or hay fever may be explained by the natural history of atopic disorders, known as the atopic march. Atopic march refers to the development of atopic eczema and hypersensitivity to food or other allergens in early childhood, and later progressing to asthma and allergic rhinitis, or hay fever, in later life [1]. The majority of children in our sample were diagnosed with the outcomes during childhood. However, age at diagnosis was by maternal or self-report, which may not be accurate. If age at diagnosis for eczema was understated and the children were diagnosed with eczema after asthma or hay fever, then there may be other mechanisms underlying the null associations between TCDD and the other atopic conditions. However, if in utero exposure to TCDD did minimize the development of eczema, this may have created a dent in the progression of the atopic march, and subsequent asthma and hay fever may not be observed.

The significant association observed for initial TCDD exposure and not TCDD estimated at pregnancy may indicate that initial body burden at the time of exposure is the relevant dose in impacting the children’s health outcomes. TCDD exposure at the time of the accident may have induced an epigenetic change in the women’s oocytes, allowing for a transgenerational inheritance of disease outcomes [59, 60]. However, we still cannot rule out that direct prenatal exposure at the time of pregnancy is also a relevant dose because our results for TCDD exposure at pregnancy do show a tendency of decreased risk of eczema, and our study’s pregnancy-TCDD levels were only estimated measurements.

Our results also show different significant associations across the three outcomes based on the case definition used. For example, we only observed a significant association for eczema in the diagnosis-based case definition. This may be due to misclassification in symptomatology, and we observed a significant result because the diagnosis-based case definition is more accurate and specific than the one that also includes related symptoms. The opposite was true for hay fever, where we observed a significant p-interaction value for the broader case definition. This may be because hay fever is more difficult to diagnose and people may not get it diagnosed because of how common it occurs. Thus, using the diagnosis-based case definition alone may have been less sensitive and missed some cases. There are some limitations to consider when interpreting these results. First, we were not able to confirm outcomes with medical records and relied on maternal or child report, so there may be outcome misclassification. However, we do not expect there to be differential misclassification or respondent bias because the interviewers and respondents were unaware of the TCDD levels and study hypotheses. Another limitation is potential residual confounding that could have impacted our results. For example, we were not able to control for the child’s delivery type because of limited available data, and a cesarean delivery has been shown to increase the risk of developing atopic diseases [44]. Lastly, although we have an accurate measure of initial TCDD levels, we estimated TCDD at pregnancy, which may lead to non-differential measurement error for prenatal exposure. This misclassification would bias our results towards the null because the degree of exposure misclassification is not related to outcome status; the same modeling approach was used to estimate TCDD at pregnancy across all outcomes and so would not explain the specific inverse finding with eczema.

This study has several strengths. First, it is a population-based prospective cohort established based on residence at the time of explosion, which allowed us to better assess causality and measure multiple outcomes related to atopy and immune functioning. To our knowledge, the SWHS cohort represents the largest female TCDD-exposed population with individual-level TCDD exposure data [36]. Also, because serum TCDD was obtained from blood samples taken near the time of the accident, we have a direct measure of the individual’s exposure level as opposed to other studies that have relied on indirect measures such as maternal dietary levels [22, 23]. Therefore, we were able to reduce exposure misclassification with biologic measurements. Another advantage compared to other studies is that exposure was predominantly to TCDD, whereas other studies examined other dioxin-like compounds including PCDD, PCDF, and dioxin-like PCBs. In our study, levels of other dioxin-like compounds were not elevated above background levels although 1976 background levels were high, compared to more recent studies [42].

## Conclusions

In summary, we found that maternal serum TCDD levels near the time of explosion were associated with lower risk of eczema but there was no relationship with asthma or hay fever among children of the SWHS second generation cohort. The results are consistent with the dysregulated immune effects of TCDD observed in both animals and human studies, and support other evidence suggesting that prenatal TCDD exposure may increase the development of infectious diseases and decrease allergic conditions. With continued follow-up of SWHS children using antibody titer data and more accurate measurements of TCDD at pregnancy, we will begin to better elucidate mechanisms underlying these findings.

## Additional file

Additional file 1: **Table S1.** Unadjusted regression models for associations of maternal 1976 TCDD and TCDD estimated at pregnancy with child atopic conditions, SWHS, Italy, 1976–2016. (DOCX 14 kb)

## Abbreviations

AhR: Aryl hydrocarbon receptor
BMI: Body mass index
DAG: Directed acyclic graph
GAM: Generalized additive models
IgE: Immunoglobulin E
IgG: Immunoglobulin G
IQR: Interquartile range
ISAAC: International Study of Asthma and Allergies in Childhood
PCB: Polychlorinated biphenyls
PCDD: Polychlorinated dibenzo-*p*-dioxins
PCDF: Polychlorinated dibenzofurans
ppt: Parts per trillion
RR: Relative risk
SD: Standard deviation
SWHS: Seveso Women’s Health Study
TCDD: Tetrachlorodibenzo-p-dioxin
TEQ: Total dioxin toxic equivalents
TH1: Type 1
TH2: Type 2
Treg: Regulatory T
