# Supplementary material for: Prenatal exposure to TCDD and atopic conditions in the Seveso second generation: a prospective cohort study
Source: Environ Health. 2018 Feb 27;17:22. doi: 10.1186/s12940-018-0365-2 (PMC5827999; doi:10.1186/s12940-018-0365-2)
Supplement: Supplementary file 1 — Table S1. Unadjusted regression models for associations of maternal 1976 TCDD and TCDD estimated at pregnancy with child atopic conditions, SWHS, Italy, 1976–2016. (DOCX 14 kb) [file 12940_2018_365_MOESM1_ESM.docx]

Additional file 1: Table S 1 Unadjusted regression models for associations of maternal 1976 TCDD and TCDD estimated at pregnancy with child atopic conditions, SWHS, Italy, 1976–2016.

|  | |  | | Unadjusted RR (95% CI) | |
| --- | --- | --- | --- | --- | --- |
| Outcomes | | Cases N (%) | | Maternal 1976 TCDD | TCDD estimated at pregnancy |
| Eczema | |  |  |  |  |
|  | Diagnosis and symptoms | 98 (14.5) | | 1.01 (0.76, 1.34) | 0.82 (0.61, 1.11) |
|  | Diagnosis only | 55 (8.2) | | 0.69 (0.46, 1.04) | 0.80 (0.54, 1.17) |
| Asthma | |  | |  |  |
|  | Diagnosis and symptoms | 152 (22.5) | | 1.04 (0.82, 1.32) | 0.85 (0.64, 1.11) |
|  | Diagnosis only | 81 (12.0) | | 1.13 (0.79, 1.61) | 0.74 (0.47, 1.14) |
| Hay fever | |  | |  |  |
|  | Diagnosis and symptoms | 243 (36.0) | | 1.04 (0.89, 1.20) | 1.14 (0.96, 1.34) |
|  | Diagnosis only | 130 (19.3) | | 0.99 (0.79, 1.24) | 1.13 (0.89, 1.42) |
